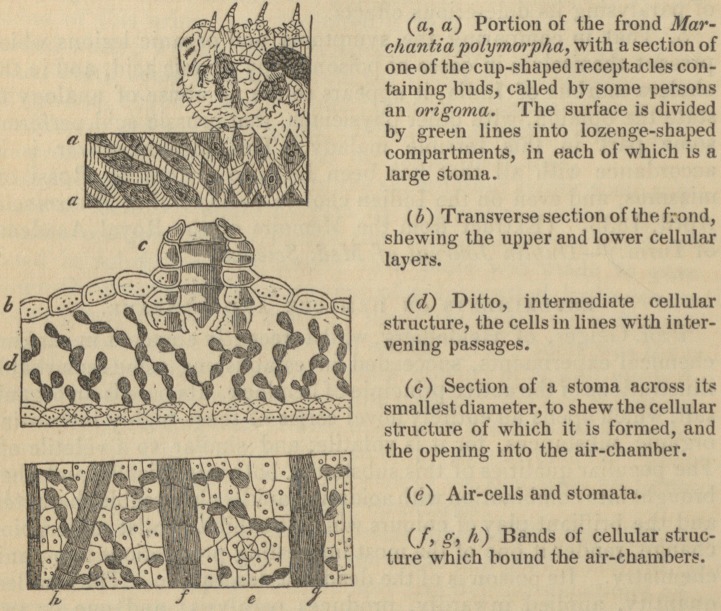# Collectanea: Miscellaneous

**Published:** 1833-10

**Authors:** 


### MISCELLANEOUS.

### THE PRESCRIPTIONS IN GÖLIS ON HYDROCEPHALUS.

Dr. Gooch, in the preface to his translation of Gölis (published in 1821,) observes, "there are some parts which I cannot satisfactorily understand; what the masked fatal intermittent is, I can neither guess nor learn; I should be puzzled to prepare the decoctions according to the formulæ given. And at page 267 he says, "The following formulæ are reprinted from the original treatise, though of some (the decoctions) the exact mode of preparation is not clear."

As it is reasonable to suppose that what perplexed so clear a head as Dr. Gooch's, must have been a stumbling-block to others, we will quote the first two of the prescriptions, and explain what seems to have been the difficulty.

Rp. Radic. althæ. alb. unciam semis.
Coq. per $\frac{1}{4}$ horæ Colat. unciarum sex.—Adde
Nitri puri drachmam semis
Syrup. ononidis
—— AlthϾ āā unciam semis.
Every half hour one or two table-spoonsful.

IV.—Rp. Rad. alth. alb. unc. semis
—— Ononid. drachm. duas
Coq. per $\frac{1}{4}$ hor. s. q. aquæ Colat. unc. sex.—Adde
Acet. ammon. solut. drachm. semis
Syrup. ononid. unc. unam.
To be taken as the preceding.

The meaning is, take as much water as after boiling and straining will amount to six ounces: the quantity necessary is not stated, but would easily be learned by one or two trials.

The "masked fatal intermittent" is probably an aggravated case of infantile remittent fever. Dr. Gooch says, "the water-stroke will be recognised by the experienced reader; it is well named, and deserved to be distinguished." Pref. p. iv. Wasser-schlag, which he translates "water-stroke," means serous apoplexy; the term is not peculiar to Gölis, but used by every German writer.

MEAT, DRINK, AND CLOTHING.

Formerly a certain sum was paid to the soldier, from which he provided himself with food; but he so often spent in liquor what he ought to have spent in meat, that it became necessary for an officer to inspect his meals. A story current among soldiers illustrates the preceding statement. An officer going round the the dinner-table of the mess, saw one without meat before him. "Donald, where is your meat?" "O, here it is, sir," showing a vessel of slop, containing a mass of something like tripe. Day after day the same appearance was presented, till the officer, having some suspicion, demanded the exposure of the meat. "O, it is tripe, sir," said Donald. "What, do you eat tripe every day? I must see it." On striking a fork into the mass, the officer continued,—"Well, Donald, I never before saw tripe with buttons on it." In fact, the meat proved to be a slice of leather smallclothes. —*The Effects of Arts, Trades, &c. on Health and Longevity. By C. T. Thackrah, Esq.*

SNAILS.

That Mecænas of cookery, Sir Kenelm Digby, who is remembered for so many odd things, was one of the persons who introduced the great shell-snail (Helix pomaria) into this country as a delicacy. He dispersed the breed about Gothurst, his seat near Newport Pagnel; but the merit of first importing it is due to Charles Howard, of the Arundel family. The fashion seems to have taken; for that grateful and great master-cook, Robert May, has left several receipts for dressing snails, among the secrets of his fifty years' experience. Snails are still sold in Covent-garden as a remedy for consumptive people. I remember, when a child, having seen them pricked through the shell to obtain a liquor for this purpose, but the liquor was as inefficacious as the means to obtain it were cruel. They were at that time, I know, eaten by the men who worked at the glass-houses, probably from some notion of their restorative virtue.

Shell-snails of every kind are rarely found in Cumberland; the large brown species I have never seen there. The snail is so slow a traveller, that it will probably require many centuries before he makes the tour of the island.—*Southey's Omniana.*

### ON THE WALK OF QUADRUPEDS.

BY J. A. BORELLI, PROFESSOR OF MATHEMATICS, NAPLES.*

Distinguished philosophers and anatomists, no less than the unlearned, have fallen into gross mistakes upon this subject, in consequence of trusting to theoretical opinion, rather than to the evidence of observed facts.

*Proposition.* The step of quadrupeds is not performed by alternately lifting the two feet diagonally opposite, while the other two remain at rest.

An opinion prevailed that the step of quadrupeds was performed by the moving forwards of two feet together, alternately with the two that are at rest, as in the walk of a man (*bipedis*) by the movement of one foot constantly succeeding that of the other. Under this erroneous impression, the ancients observed that quadrupeds, while they stand upon the four soles of their feet, form upon the ground a four-sided figure, A B C D. It was thence remarked, that when they are at full speed, the two fore feet, A B, are raised and moved forward together, whilst both the hind feet, C D, are at rest. Immediately after A B falls to the ground, the two hind feet,

* Translated from the Latin work "De Motu Animalium," by John Sharp, esq.; and extracted from the "Field Naturalist's Magazine."

C D, are raised and moved forwards near to A B; and in this manner quadrupeds, by successively contracting and lengthening themselves, perform running, as is evident in the case of horses and dogs at full speed.

But, in going at an easy pace, it is evident that the two fore feet or the two hind feet are not raised together, nor moved forwards at the same time, but alternately; when A is moved, B is at rest; and, on the contrary, when B moves, A is at rest. It is certain that this takes place in the hind feet: but it cannot be so easily distinguished in what order the fore feet move with the hind feet, I mean whether the two left feet, A and D, are moved at the same time, or A with C, on account of the quickness of the motion.

They were of opinion, however, that this could be ascertained by a process of reasoning. If the two left feet, A D, were raised and moved forwards at the same time, then the animal would fall upon the left side. Therefore, the right fore foot, B, with the left hind foot, D, ought rather to rise and move forward at the same time, so that the feet diametrically opposite might move or rest together. Moreover this erroneous opinion prevailed to so great a degree, that, in equestrian statues of marble and of bronze, both ancient and more recent, the two feet diametrically opposed are suspended from the ground.

I am truly surprised that the difficulty and absurdity of such a notion was not perceived. They grant that an animal ought to be steady in motion, lest it might totter or fall, and hence they aver that the two left feet, A and D, could not be moved together; for then the centre of gravity of the quadruped, and the line perpendicularly drawn from it to the ground, would fall either upon the same right line, B C, where the two right feet are at rest, or beyond it on the line A D, and in that posture the animal would totter or fall.

But when the two feet, B D, diametrically opposite, are raised and moved together, at the same time, the whole weight of the animal ought to rest upon the two feet fixed upon the ground; I mean the line perpendicularly drawn will fall not upon the large space, but upon the line A C. The animal would therefore equally totter; and thence it will have an insecure and unsteady posture at that time.

Secondly. We may consider the figure, which the four feet form after the first motion—namely, when the foot B is transferred to K, and D to S; then the two left feet, A and S, become contiguous, and the right feet, K C, in turn, are most removed from each other, so that the four feet form a triangular figure, the longest side of which is K C, and the least altitude A B. This posture therefore is not secure enough, and from it, after the motion of the feet, C and A, to I and V, the animal is restored to the steady quadrangular position, I S V K, like the former A B C D. The firm and tottering postures of this kind regularly following each other would have been unwisely ordered by Nature, when these disadvantages could have been easily avoided.

But why do we inquire for reasons, when experience contradicts the facts inferred. Observe a horse moving at a slow space, and you will never see the two feet, A and C, diametrically opposite, to move at the same time, but one foot is always raised from the ground while the other three are at rest. By an attentive observation, you will afterwards perceive that this process is followed in the quick motion of all kinds of quadrupeds.

[In birds, as we shall see in a future page, there are two species of this sort of movement; one group, like the nightingales and sparrows, carrying both legs forwards at the same time, or hopping; another, like the wagtails and the blue-breast (*Motacilla Suecica*), putting one foot before the other. Edit. of F. N. M.]

The manner in which the step of Quadrupeds is performed, explained.

The oblong figure of a horse, which may be considered as resting upon its legs as upon pillars fixed upon the ground in A B C D, forms a four-sided rectangle; the line drawn perpendicular from the centre of gravity of the horse would fall upon E near the centre of the rectangle, and thus the posture of the animal would be most secure. Afterwards the step is made by the foot behind, as the left, C, which, by pressing the firm ground with a strong effort backwards, the centre of gravity is moved forward from E to G, and this being performed quickly, the foot B is raised and moved forwards to H, which motion can be made with greater effect, because the centre of gravity falls at first within the triangle A B D; next, it falls within the four-sided figure (*trapezium*) A B F D, that is, it is supported upon three or four columns. The next three feet, A D F, remain firm, and include the centre of gravity G; the left foot B, being moved forward to H, and by this impulse the centre of gravity is transferred to I, that is, to the centre of the four-sided figure (*rhombus*) A H F D; the steps of the two left feet being concluded, the motion of the right hind foot D begins, and afterwards that of the fore foot A. The step of quadrupeds is always performed in the order just explained.

Although attentive observation may be sufficient to prove these facts, yet it is the duty of the philosopher to inquire into the advantages and necessity of such a process. It is an invariable law of Nature, while she avoids as much as possible the disadvantages and complexities of any system, to perform her work by means sure and certain, and at the same time the easiest and most simple.

The step of animals does not include the motion of the whole body moved forward, with equal rapidity and in the same direction, as in flying, leaping, or creeping, but it is rather the motion arising from the transference of some parts of the body, which are supported by others at rest, and in this manner an animal is moved forward in walking, standing and moving by turns. For this reason, the standing and moving, which includes the step of animals, cannot be tottering and unsteady, but firm and secure, and ought to be performed with the least yet sufficient labour of the muscles. But as the attitude of a quadruped in walking must be free from the risk of falling, it is necessary that the body of the animal be supported upon more than two columns, I mean upon three or four, within which the line perpendicular to the centre of gravity may fall. It is very evident that the step of quadrupeds is performed in the manner explained in this proposition.

It is moreover necessary that the support of the animal should be accompanied with the least labour and pain to itself, and this is accomplished by the legs serving as columns, which, on account of their hardness and obtuseness of feeling, easily support the superincumbent weight, without any remarkable uneasiness.

Besides, the motion of the animal body is easily performed, because its whole mass is not raised from the ground at once, one foot only being lifted and moved forward. This is performed by first pressing upon the ground with one of the hind feet, and not by one of the fore feet, for if the former was lengthened while the latter rested upon the ground, the centre of gravity would fall behind; but, on the contrary, by one of the hind feet being lengthened, the fore foot is moved forwards like a gladiator's spear (*ad instar conti*); whence it happens that the whole mass of the animal is moved forward by the bending of three erect columns, not different from the manner in which running upon wheels is performed. Next, by raising from the ground the same hind foot, the joints being bent by the muscles, afterwards by the motion of the fore foot of the same side as has been mentioned, these advantages, I say, show the necessity of such an operation.

### THE GUNNER WITH THE SILVER MASK.

Alphonse Louis, aged twenty-two years, a native of St. Laurent, in the Pas de Calais, private in the 5th company, 2d regiment of artillery, of a sanguine temperament, was wounded in the trenches on the 6th December, 1832, by the splinter of a shell. When this misfortune occurred, Louis stood fronting the left wheel of his gun. He held a lever or handspike across his body, in the proper position of a gunner waiting to serve his piece; that is, with the right hand raised and the left depressed, or the very reverse of the position of an infantry officer when holding his sword diagonally, at open order, preparatory to a salute. At this moment a twelve-inch shell burst a few feet above the battery, and a fragment of about seven pounds weight struck Louis.

The projectile first attacked the external part of the left jaw, carrying away almost the totality of the maxillary process, of which there only remained the edge of the extreme left posterior portion, the coronoid process, and condyle. On the right side, the extremity of this bone was preserved as far forward as the first large molar tooth, inclusive. Besides this, the alveolar processes and teeth of the upper left jaw were partly fractured, the body of the hyoides laid bare, the left parotid duct lacerated, and the tongue furrowed on the same side with a deep wound.

The loss of substance, or solution of continuity, occasioned by this wound, was immense; it extended on the left side from the zygomatic process to the anterio-superior articulation of the thyroid cartilage, tearing away almost all the fleshy parts of the cheek, and a large portion of the upper lip; on the right side it ran from the same cartilage to a level with the upper maxillary sockets and the buccinator, to within half an inch of the lobe of the ear. The centre of the wound was occupied by lacerations of the roof and coating of the palate, by the œsophageal opening of the throat, by some remnants of the upper maxillary glands, and the hyo-gloss and genio-gloss muscles; and lastly, by the tongue, swollen to four times the size of its ordinary volume. This organ having been entirely denuded on its lower superficies, as far as its base, and having lost its natural support, hung down in front of the larynx. In short, to render the description still more clear, nothing whatever remained of the lower jaws save the four fractured double teeth, and injured fragment on the right side, and thus the tongue drooped down to the length of several inches, exposing the cavity of the throat,—a horrible and ghastly sight.

But the sufferings of the victim did not terminate here; the splinter, after committing this fearful havoc in the face, encountered on its descent the up-raised right arm, and striking it about one third of the distance from the elbow to the wrist, caused a compound fracture of the severest kind.

Louis was immediately raised, and carried from the battery to Hoboken, where the surgeon-major on duty forthwith proceeded to sew up several portions of the integuments of the upper maxillary region, as well as those of the neck; that is, both above and below the solution of continuity, in order to diminish the aperture. But the nature of the laceration was such, as to offer little hope of saving the patient; indeed, such was the nature of the mutilation, that death appeared not only inevitable, but ardently to be desired. The amputation of the fore-arm was then performed by the ordinary process, at the distance of about two inches below the articulation of the elbow-joint. The usual dressings were applied in both instances. Symptoms of general re-action were not long in developing themselves, at first not with a degree of severity proportionate to the gravity of the wounds. The most rigorous diet was enforced. The wounds of the face, fearfully swollen, were dressed every day without any remarkable accident; and on the 11th of December, or sixth day, the sewings and first dressing of the fore-arm were removed; and on the 12th the patient was carefully transported to the reserve field-hospital at Boom. On the following days the same treatment and regimen were continued, and Dr. Forjet was not without hopes of saving the man's life.

The suppuration now commenced in both wounds: it was of a satisfactory nature as regarded the stump of the arm, but of a less favourable character in the face, of which several mortified portions gradually sloughed away. Between the fourteenth and twenty-fourth days, the gashes in the face assumed a livid and gangrenous character; the suppuration was more unwholesome, and the dressings more painful than ever. The stump having become the seat of considerable inflammation, the bandages were removed, and emollient cataplasms applied: notwithstanding this, however, the extremities of the bone pierced through the fleshy parts near the lips of the wound, and their death was the inevitable result.

On the 2d of January, or twenty-eighth day, the patient was transported from Boom to the military hospital at Antwerp, and placed under the immediate care of Drs. Forjet and Seutin.

From the 2d to 20th January, the dressings could only be effected by the aid of the chloruret of sodium: these were renewed daily. But, notwithstanding the most energetic treatment, and the most indefatigable attention, the aspect of the wounds was extremely unpromising, and nothing could arrest the gangrene. However, between the 15th and 20th, the surfaces assumed a less unfavourable appearance, and the local and general symptoms were attended with a slight amelioration. This amelioration progressively advanced, in consequence of the unremitting care with which the patient was attended. The nourishment at first consisted of some slight doses of thin broth, afterwards veal soup, and lemonade tinged with wine, and then with vegetable and animal jellies. In proportion as the muscles of the tongue regained some slight power the deglutition of these substances became less laborious and painful. They were administered by means of a narrow curved spoon, moulded for the purpose, the extremity of which was placed on the base of the tongue, and the food, always administered in a liquid state, poured down the œsophagus.

From the 25th January to the 9th February, the progress of general and local amendment became more sensible daily, the edges of the wound subsided to a level with the circumjacent surface, and the work of cicatrization manifested itself. The tongue was now reduced to within double its ordinary volume, and the exfoliation of bony substances continued insensibly. The cicatrization of the stump was only impeded by the complication before mentioned. The portion of dead bone came away on the 9th; other fragments had been previously removed, and the whole appeared in a promising state.

A singular circumstance attended this part of the case, and merits peculiar attention. Although the gustatory surfaces were diminished by nearly three fourths, and the tongue had lost its action, although alimentary substances only came in contact with a small part of the posterior portion of the tongue, and the mucous membrane of the bronchial passage (which generally are not admitted to possess the faculty of distinguishing or appreciating the savour of any given nutriment, but merely that of facilitating deglutition,) it appeared that the sensation of taste, though [illegible] first much impaired, was now exercised with perfect discernment.

On the 10th February an exact plaster cast was taken of the face, thus horribly disfigured; it being sufficiently cicatrized to permit this operation without causing great suffering to the patient. A cast-iron mask was then made; and by the aid of this the artist was enabled, at his leisure, to construct a substitute for the lost parts, that might not only render essential service to the individual, but even deceive the eye as to the ravages of the wound. From the 10th to 25th the process of cicatrization made rapid progress. The palate, uvula, the whole of the injured superior surface and adjacent membranes, had returned nearly to their natural state, and the phenomena of deglutition gradually increased in the regularity of their functions; however, the extent of the vault of the palate was still much diminished, and its surface covered with a thick white crust, demanding constant ablution. The left parotid was paralyzed, and the salivary process only went on by the right side. On the other hand, the stump was in a highly satisfactory state, and almost entirely cicatrized. The general condition of the patient was, in every other respect, that of the most promising convalescence.

The integrity of the vocal organs having remained unimpaired, it is unnecessary to observe that the voice continued unchanged: this however was not the case with the powers of articulation, which could not of course act, from the want of the front of the mouth. It is not however uninteresting to remark, that the phenomena of speech were not entirely annihilated; that the simple and compound vowels were uttered naturally, and that the greater part of the consonants were pretty distinctly enunciated; the labial and hissing sounds were the most indistinct. Nevertheless, the mere habit of attending to the patient sufficed to render his language intelligible, and an improvement in his mode of pronunciation was gradually perceptible; so much so indeed, that there was every ground for predicting that the modifications in his power of articulation would be susceptible of great improvement, under the influence of time and education. This prediction has already been partially verified; for, on our last visit to Alphonse Louis, we were able to comprehend every word he uttered; and the wounded men occupying adjacent beds in the hospital said that they understood him as well as any other of their comrades. He appeared to speak without any great effort or pain. The sounds were not loud, but sufficiently so to be heard at a reasonable distance; and gave one the idea of a person speaking with a wooden gag placed transversely between the open jaw, and pressed against the back of the mouth.

On the 25th Dr. Forjet made some attempts to separate the morbid fragments of the right jaw, and succeeded in detaching the whole portion of the bone that incommoded the patient, and prevented the precise application of the artificial substitute. The rest of the operation was left to nature; and four days subsequently the splinter separated itself from the live parts. It consisted in front and below of the whole thickness of the maxillary body; and above, of the bone that separates the 5th and 6th dental socket. On the outside it was composed of the extreme portion of the body and branch of this bone, including its obliquity, terminating backwards in the coronoid process. The patient still preserved on this side the three large molars, of which the first, imbedded in a half socket, forms the antero-posterior base of the maxillary bone, and might resist for a length of time the ordinary causes of destruction.

It was at this time that the last finish was given to the artificial silver substitute, the execution of which had been intrusted to a skilful artist of Antwerp, M. Verschuylen, from the designs of Dr. Forjet. The ability shewn by the artist in the construction of this ingenious piece of mechanism, is deserving of much commendation; and we venture to recommend such of our readers who may have an opportunity of visiting the Invalids at Paris, to inquire there for the "Gunner with the Silver Mask;" they will then be enabled to inspect the contrivance, and to see and converse with its proprietor. To practical men the visit will be of deep interest. A short description of the mask itself is necessary, though we cannot pretend to offer a graphic portrait.

The external part is composed of a lower half-mask, without nose or cheeks. The anterior edges are in immediate contact with the lower part of the nasal cartilage and adjacent muscles, and the angles of the upper jaw. The two sides, or half-cheeks, repose on the parotid borders of the maxillary and the sterno-mastoideum, so as to conceal and enclose the whole extent of the deformity. In the front of its centre, that is, the portion occupied by the lips and chin, there is an oblong square plate, or trap, opening with a lateral hinge and spring; this imitates the surface of the chin, two lips, and middle section of the mouth. This trap being opened by the patient's left hand, shews a second, or internal chin, and complete local cavity, with a regular set of metal teeth. By the aid of this aperture, of which the mechanism is extremely simple, a communication is opened between the air and the pharynx, so that he can repose and breathe freely without taking off his mask. This is not strictly necessary for the process of respiration, as there is an opening between the artificial lips; it is merely done to give greater freedom to the action of the lungs, and to diminish the heat.

All the points of contact with the face are skilfully ornamented with mustachios and whiskers, which entirely cover the edges. The inferior parts are covered by the cravat; and the posterior part, which reaches behind the ear, hidden by allowing the hair to grow and fall down over it. At the distance of two or three yards it is impossible to distinguish the artificial nature of the substitute; the subject having the appearance of a man of good constitution between forty-five and fifty years of age. The mask is painted in oils, of a tint analogous to his complexion, so that the illusion is so strong that, unless forewarned, he might be stedfastly examined at a short distance without betraying his misfortune.

The internal part is divided into two compartments. The upper, or sublingual section, is furnished with a platform which supports the tongue, retains it in its proper position, and regularly circumscribes its action by a complete alveolar process, set with gold teeth. This jaw, being adapted with a hinge and spring, can be lowered at will by the man's left hand, so as to admit food. The lower section forms the cavity of the inward chin, and is disposed so as to serve as a reservoir for the saliva and mucous secretions, which are incessantly flowing from the remaining parotid and glandular integuments of the mucous membrane; these fluids are got rid of through a small orifice, by merely leaning the head to one side. The different portions of the mask are of silver, strongly gilt, and so constructed that they can be taken to pieces in order to undergo cleansing, and can be re-united with the utmost facility. The whole contrivance, an admirable proof of mechanical skill, is maintained in its proper place by means of Indian rubber bandages, which hook on the occiput and vertex, and are strengthened by means of a flexible metallic strap, intended to prevent all possibility of derangement. The weight is about three pounds, and the cost of the whole was about 12*l.* sterling.

The use of the mask is by no means painful or inconvenient, considering the nature of the wound. It is, above all, of great assistance in arresting in their passage, and retaining in the cavity of the artificial chin, the salivatory and mucous secretions; it facilitates the action of the tongue; it has restored a face dreadfully mutilated to a human form; it has singularly softened the rigour of the sufferer's fate, conduced to his comfort, and rendered his existence not only desirable, but comparatively happy. On our last visit to Alphonse Louis, the day previous to his departure for Lille, he appeared in high spirits; he walked about with agility; used the stump of the fore-arm with address; took off and readjusted his mask with his left hand; spoke not only intelligibly but easily; he was high-coloured, and fatter, as he stated, than he had ever been prior to his misfortune. He played at cards, and seemed to be as proud of shewing the mechanism of his artificial jaw, as he was of the crosses of the Legion of Honour and Leopold, that glittered on his bosom.

It would be in our power to detail several other most interesting operations performed by Dr. Forjet during the siege, but we have already occupied too much of the reader's time with the description of the "Gunner with the Silver Mask;" whose history, though less romantic than that of the celebrated "Iron Mask," is infinitely more interesting to science and humanity. Of the one, all that can be said is, that he was the mysterious victim of that most unparalleled despotism that paved the way to the first French revolution; in the other, we have a proof of the glorious triumph of art, and of the immense progress of medical skill. Wounds of a similar nature are not unfrequent; there is an instance, we believe, of something analogous in the case of Colonel Cunningham; but, as far as we have been able to ascertain, no recovery from such complicated lacerations ls on record in France, nor had the substitution of an entire artificial jaw ever been projected, or successfully executed, until the present instance.—*Medical Gazette.*

### M. QUETELET ON THE WEIGHT OF MAN AT DIFFERENT AGES.

The facts in this paper are curious and valuable, both with respect to physiology and forensic medicine. All the researches hitherto made on the subject have had reference to the period of birth, or to the epoch of complete development :-little has been done for the intermediate ages. But the great importance of the inquiry relative to the progressive development of man, is evident from the problem, which is often proposed to the medical jurist—to state the age of an individual from an examination of his physical properties. An opinion may be hazarded by an inspector, and his report may be received; but, unless he have taken into account the stature and weight of the individual, as well as certain other peculiarities capable of measurement, his opinion is a mere dictum; vague, and destitute of any solid support. The height of man has been frequently examined: the weight it has remained for M. Quetelet to inquire into, with all that exactness of which the subject is susceptible.

As to *new-born infants*, from observations made on 63 males and 56 females, in the Maternité de Saint Pierre, it appears that the mean weight of the former was 3·20 kilogrammes (6·536lbs.), while the length, by Chaussier's mecometer, was 0·496 metres (1 foot, 6 inches, 3 lines); and of the females, the mean weight was 2·91 kilog. (5·923lbs.), the length 0·483 metres (1 foot, 5 inches, 10 lines.) Whence it is inferred that at birth there is an inequality in the weight and size of the two sexes, the males having the advantage in both.

Chaussier seems to have been the first who remarked that the infant, presently after birth, begins to lose some of its weight. M. Quetelet, from seven series of observations, extending in each case to the seventh day, has confirmed M. Chaussier's remark, and shews that the infant does not begin to grow perceptibly till after the first week.

M. Quetelet gives a table of the corresponding weights and statures at the different ages. We extract a few of them, by way of specimen:

| Ages. | Males. | | Females. | |
|---|---|---|---|---|
| | Height. | Weight. | Height. | Weight. |
| | *m.* | *k.* | *m.* | *k.* |
| At Birth | 0·500 | 3·20 | 0·490 | 2·91 |
| 1 | 0·698 | 9·45 | 0·690 | 8·79 |
| 3 | 0·864 | 12·47 | 0·852 | 11·79 |
| 6 | 1·047 | 17·24 | 1·031 | 16·00 |
| 10 | 1·275 | 24·52 | 1·248 | 23·52 |
| 20 | 1·674 | 60·06 | 1·572 | 52·28 |
| 30 | 1·684 | 63·65 | 1·579 | 54·33 |
| 43 | 1·684 | 63·67 | 1·579 | 55·23 |
| 50 | 1·674 | 63·46 | 1·536 | 56·16 |
| 70 | 1·623 | 59·52 | 1·514 | 51·51 |

### THE CONSTITUTION OF POPE.

Headach was the urgent symptom which Pope constantly complained of, and this he was in the habit of relieving by inhaling the steam of coffee. It is difficult to conceive on what principle this remedy could alleviate his sufferings; but, from the manner in which he aggravated them by improper diet, it is very probable that his remedy was no better than his regimen. It appears that, like all dyspeptic men, he was fond of every thing that was not fit for him. "He was too indulgent to his appetite," says his biographer; "he loved meat highly seasoned, and if he sat down to a variety of dishes, he would oppress his stomach by repletion; and though he seemed to be angry when a dram was offered him, he did not forbear to drink it: his friends, who knew the avenues to his heart, pampered him with presents of luxury, which he did not suffer to stand neglected. We are told by Dr. King, his contemporary and friend, that his frame of body promised any thing but long health, but that he certainly hastened his death by feeding much on high seasoned dishes, and drinking spirits."

From the various accounts given of his mode of living, and of the sufferings it entailed on him, it was evident that his appetite was depraved by indigestion; and it is no less obvious, that constitutional debility induced by that deformity, either natural or accidental, under which he laboured from his cradle, had given the predisposition to this disorder. His frequent heahach s, and the sensation of confusion and giddiness after application to study, or excess in diet, those premonitory symptoms of dyspepsia, he appears to have looked upon as his original disease, whereas the stomach was the seat of his disorder, and the affection of the head only sympathetic with it. Yet it must be admitted, that when literary men are the subjects of this disorder, that it is very often exceedingly difficult to determine whether the head or the stomach is primarily affected; but in whichever of them is its origin, so immediate is the influence of the one on the other, that the treatment is not materially embarrassed by our uncertainty of the primary seat of the disease. It is the nature of parts sympathetically affected to become disordered in their functions, rather than organically diseased; at least it is a considerable period before any alteration of structure in a symptomatic disorder takes place. The interval between the two results is occupied by a long train of anomalous ills, which are generally denominated nervous. The term is vague and unmeaning enough for all the purposes of nosology. It implies a host of sufferings, which sap the strength and sink the spirits of the invalid, and this hydra-headed malady may continue for years an incubus on his happiness, which utterly destroys not health, but renders valetudinarianism a sort of middle state of existence between indisposition and disease. The symptomatic affection of the head only becomes an organic disease, when the long-continued cause has given it such power that the effect acquires the force of a first cause in its influence on an organ previously weakened or predisposed to disease. It is then easily conceived how the simple headach, in the case of Pope, continued for years symptomatic of a disorder of the stomach, aggravated by mental excitement and improper diet, till the disturbance of the functions of the brain ultimately debilitated that organ, and left it no longer able to resist the effects of the constant exercise of the mental faculties. The result of such long-continued disturbance of the cerebral functions, there is generally great reason to apprehend, will be either alteration in the structure, softening of its substance, or effusion serous or sanguineous.

There is great reason to believe that one of these terminations took place in the case of Pope several years before his death, as it was found to have done in the case of Swift, and more recently in that of Scott. Even when Pope was apparently in the enjoyment of tolerable health, he had evident symptoms of pressure on the brain, or at least of an unequal and imperfect distribution of the blood in that organ. Those symptoms are only noticed by his contemporaries as curious phenomena connected with his habits of life. Spence says he frequently complained of seeing every thing in the room as through a curtain, and on another occasion of seeing false colours on certain objects. At another time, on a sick bed, he asked Dodsley what arm it was that had the appearance of coming out from the wall; and at another period he told Spence, if he had any vanity, he had enough to mortify it a few days before, for he had lost his mind for a whole day. Well might Bolingbroke say, "the greatest hero is nothing under a certain state of the nerves; his mind becomes like a fine ring of bells, jangled and out of tune!"—*Madden's Infirmities of Genius.*

### ON DISSECTING AND PREPARING ANIMALS FOR COLLECTIONS. BY PROFESSOR CARUS OF DRESDEN.

Though the art of anatomising the bodies of animals is essentially the same as that practised upon the body of man, and though want of space precludes me from treating the subject minutely, I conceive that a few remarks may not be altogether unacceptable to those who feel desirous of pursuing such studies for themselves.

The first thing that I have to observe is, that all dissections of small and soft objects, such as worms, zoophytes, insects, mollusca, and embryos, where it is desirable to obtain even tolerable accurate results, should be performed under water, by which the parts are kept floating and separated from each other, and consequently present themselves more distinctly.

A very simple contrivance for investigations of this kind may be prepared in the following manner: A mass of tough wax (not too soft) is to be laid upon one or more porcelain saucers or capsules of different sizes, which are then to be put in a warm place until the wax melts so as to cover the surface evenly to the depth of a half or a third of an inch. If the object to be examined be laid upon this surface, it may be fixed by needles in any position that is wished, and, when covered with clear water, developed and dissected by means of suitable instruments. Of these the best are very delicate forceps; pointed, well-made, sharp-cutting scissors; and small knives, like cataract needles, some round, others with cutting edges, and fixed in slender wooden handles. For separating parts I have also employed small horn probes and fine brushes; whilst, for examining them, a good magnifying glass is frequently indispensable. If it is wished to preserve a preparation thus made, wax, coloured at pleasure as for the purpose of injections, is to be formed into little tablets about one-fourth of an inch thick; one of these is then to be placed upon the saucer or capsule containing the preparation the latter may then be transfered to it, arranged suitably upon it, fixed there by means of short needles, and both together then placed in alcohol. Nor must I forget to mention, that the examination of very delicate organizations may frequently be conducted with greater facility and accuracy if the object be previously allowed to remain some time in spirit, and thereby to become harder and contracted. This applies particularly to the dissection of nervous organs, and to the examination of very small embryos, of mollusca, and worms. There are various methods of destroying worms, insects, mollusca, &c., for the purpose of dissecting, without injuring their organization: mollusca, snails, for instance, as Swammerdam has remarked, are to be allowed to die in water, because by that means their body swells, and all the parts beeome more distinctly visible; they may be afterwards kept in spirit (though not too long) for dissection. Worms, the larger zoophytes, (for the smaller must be examined while alive,) caterpillars, &c., and also the smaller amphibia and fishes, are best destroyed by means of spirit; insects, on the contrary, by being dipped rapidly in boiling water, or in oil of turpentine. As regards the dissection of larger animals, we may here use with advantage knives of a larger size; and, instead of forceps, suitable hooks with handles.

In animals of considerable size we can generally make artificial skeletons only after the bones have been sufficiently cleaned by boiling or maceration. In smaller animals, on the contrary, such as birds, amphibia, and fishes, of which last it is very difficult to make good skeletons, the object will be best accomplished by at once making the bones as clean as possible without injuring the capsular ligaments, soaking the preparation in water that is incessantly changed; and lastly, bleaching it for some time in the sun. Lastly, we may mention injections as affording a very essential assistance in zootomical investigations for physiological purposes. In small animals, and in the most minute parts, these must consist of compositions with wax, very fluid and coloured; best of all of mercury. The latter, however, is not suitable for very soft bodies, such as medusæ, &c., in which cases we may employ injections of coloured milk, and similar substances.—*Translated by Mr. Gore, of Bath, in the Field Naturalist's Magazine*, No. II.

### COMMERCIAL TRAVELLERS.

Few commercial travellers bear the employ for thirty years, the majority not twenty. Thus an occupation, in itself so healthy that a man might follow it from boyhood to eighty in health and vigour, is corrupted to the production of disease, and the destruction of at least half the term of human existence. I attended a commercial traveller who pursued his employ to the age of seventy-nine. His habits, however, were widely different from those of his class.

I am favoured by Mr. Pierce with specimens of innkeepers' charges to commercial travellers in 1774 and 1791, from which it appears that, though the diet was generally better, customers were then treated with spirits and wine, and the quantity drunk in such business-conviviality, was sometimes as great as any modern excess.

February, 1774: "Breakfast (bread and milk,) 2*d.*; Geneva, for customers, 1½*d.*; oats, half a peck, for horse, 4*d.*; hay, 1*d.*; hostler and waiter, 2*d.*"—December 9th, 1791: "Breakfast, 8*d.*; dinner, 8*d.*; tea, 8*d.*; supper, (chickens and asparagus,) 8*d.*" December 10th and 11th, the same routine of meals, at 8*d.* each, with the addition of liquor for customers, 2*s.* 0½*d.* the first day, and on the second and third days, 6*s.* 6*d.*: sums which, in those days, would supply enormous potations.—*Thackrah on the Effects of Arts, Trades, &c. on Health and Longevity.*

GEOLOGICAL DISTRIBUTION OF THE MUSCI.

(816.) Five fossil species of Chara, and one, or at the most two, fossil mosses, are all that have been as yet discovered. No vestige of a fossil liverwort has hitherto been found. It is true, as

A. Muscites Tournalii, reduced. B. Portion magnified. C. Hypnum riparium. D. Portion magnified. E. Leaf separated. F. Muscites (?) squamatus. G, H. Portions magnified. I. Sphagnum compactum. J. Separated leaf of ditto. K. Branch of Juniperus phœnicea.

Brongniart observes, that Daubenton believed he had recognised a variety of these plants in the moss-like markings of Agate; and Mr. M'Culloch published, in the Geological Transactions, several figures having a very strong resemblance to Jungermanniæ. But Brongniart, after much laborious investigation, concludes that they are simply infiltrations, accidentally assuming forms which, without very attentive examination, might be mistaken for vegetable impressions.

(817.) That the stoneworts should be found in a fossil state from the first epoch of their existence, might be presumed from the dense incrustation with which they surround themselves; petrifaction being to them a natural process, that terminates their life. But, although abundant in the beds above the chalk, their first appearance is in the lower fresh-water formation. The fossil nucules of the Chara, [§ 786, K, L,] were called by Lamarck *Gyrogonites;* but later naturalists considering the fossils to be remains of plants identical with those now existing, they of course must have the same denomination. The stems and fruit of fossil Charæ are very common in this country; and beautiful specimens are procured from the Scotch marl, and from the lakes in Forfarshire, where they are most abundant.

(818.) Of the two species of Muscites, one only is absolutely decided to be a fossil moss; the other, which at first was thought to be a Lycopodium, although now called a Muscites by Brongniart, has a query attached to its generic name.

The Muscites squamatus, [fig. E, G, H,] has long been known under the name of Lycopodites, as occurring in the mill-stone quarries near Paris.

The Muscites Tournalii, [fig. A, B,] has been but lately discovered by M. Taurnal, near Narbonne, in a fresh-water formation, consisting of chalk marl, and forming part of the tertiary series.

The former, Brongniart considers to bear a stronger likeness to the Hypnidæ than to any other mosses, and he gives a figure of *H. riparium,* [fig. C, D, E,] to shew the similitude of the leaves; at the same time pointing out various other resemblances to other species, such as *H. ripariöides*, *cuspidatum*, *denticulatum*, and *elegans*. The latter more doubtful fossil he likens to the *Sphagna*, some of the fragments bearing the greatest resemblance to *S. compactum*, [fig. I. J,] and others to *S. squarrosum*. The regularity of the four ranks in which the leaves of the Junipers are arranged, [fig. K,] will at once separate them from these remains, with which otherwise they might be confounded.—*Prof. Burnett's Outlines of Botany.*

### HABITS OF MERCANTILE MEN.

Mercantile custom, in reference to hours, varies, I believe, with place and kind of business; but in towns devoted to trade and manufactures, the application is generally excessive. It may not be improper to adduce an example or two of the cases which present themselves to a medical man. Mr. —— complains of habitual pain in the head, with occasional attacks of severe throbbing, depression of spirits, broken rest, impaired digestion, and a torpid state of the bowels. He is a slight active man of thirty, and has been in a merchant's establishment since he left school. He rises at five o'clock, or soon after, and immediately enters the warehouse, which adjoins his house. At eight he steps home to breakfast, but returns again in fifteen or twenty minutes, and is at business till half-past one. He then goes to dinner, eats it hastily, rarely sits ten minutes afterwards, but proceeds to the warehouse. Tea and supper are uncertain, and one or other is taken as convenient. The counting-house is closed at nine or ten, and he remains with the clerks to the last. Such is his general routine for five or six days a week.

Mr. —— complains of a deranged stomach, with a morbidly-recurring appetite, occasional acidity, and lately vomiting; pains in the various parts of the trunk, and defect in the action of the intestines, depending, it appears, on fault in the secretion of the bile. On inquiring his habits and circumstances, he says, "I have long been an invalid. Within the last six years I have had great losses; and my mind, of course, has been constantly hurt by finding my property slipping from me. I have worked hard for it in early life. We were at business early and late. My father used to say, 'quick at meat, quick at work;' and I have seldom allowed myself more than a quarter of an hour for any meal. I do not think, however, that the exertion of my early life has injured me so much as the anxiety and grief of later periods." Relations like these might easily be multiplied. In such cases we cannot be at a loss for the causes of impaired health, and shortened life.—*Thackrah on the Effects of Arts, Trades, &c., on Health and Longevity.*

*On the simultaneous Presence of* Prussian Blue, *and of a* Saccharine Matter, *in a particular Variety of* Human Urine. By M. Cantu, Professor of General Chemistry applied to the Arts, in the University of Turin.

The presence of prussic acid in human urine, secreted during a morbid state of the animal economy, was already announced upwards of forty years ago by Brugnatelli; and MM. Moyon and Julia-Fontanelle observed a few years back that of prussian blue and saccharine matter.

As the knowledge of this extraordinary fact may give rise to new observations and farther experiments on the part of physicians and chemists, and influence the progress of the theory and practice of medicine, this subject seemed deserving the attention of the Royal Academy.

I shall briefly state the experiments which I made on this subject, the results I obtained, and the inferences which I consider it warrantable to deduce from them.

The urine in question was sent me by Dr. Bernetti, member of the College of Medicine. According to the intimations which he had the kindness to communicate to me, it came from a little girl about eight years old, who complained of no indisposition, except some colicky pains occasionally felt in the epigastric region, a little before she experienced a necessity to void urine. It is necessary to remark that she was not at that time subject to any medical treatment, and that she used nothing but ordinary food and drink, it being merely the extraordinary circumstance of the blue colour of the urine which excited the attention of the parents, and made them consult the physician on so strange an occurrence, from which they dreaded, not without reason, some serious results.

This urine immediately when passed was of a blue colour, similar to that of the solution of indigo in dilute sulphuric acid, at least such was the colour of the urine voided at night; that passed by day was of a less deep colour; it bordered slightly on green, merely because being more watery it contained less prussian blue, on which its blue colour depended, as we shall see presently; scarcely could the odour and taste of ordinary urine be distinguished in it, but there was clearly perceived the odour of syrup of sugar, and a decidedly sweetish taste, similar to that of the urine in *diabetes mellitus*.

A portion of this urine put into an open vessel, and left to the action of the air at a temperature of from 13° to 18° Reaumur, began to assume a less deep shade after about twelve hours, it then became greenish, and finally acquired a yellow citron colour, throwing down some flocculi of mucous matter of the same colour. In the meantime, whilst these changes were becoming manifest, there was developed a slight ammoniacal odour, and re-agents indicated the presence of an alcali.

From these phenomena it may be inferred that the urine experienced a partial decomposition which gave rise to the ammonia: this latter, by decomposing the prussiate of iron contained in the same urine, dissipated its blue colour. The urine thus deprived of its colour, and altered in its nature, still left to the influence of the same causes, lost gradually the ammoniacal odour, and in the space of two days acquired a sour smell, slightly alcoholic, and its blue colour appeared somewhat less intense than before.

Hence it appears that the farther decomposition of the urine gave rise to the formation of acetic acid, and to some alcohol; and that the ammonia, having been saturated by this acid, in proportion as it was formed, the prussic acid reacted on the oxide of iron, and produced by a natural consequence the blue matter of the urine, or the prussian blue. The blue urine, not altered, put into a well-closed glass vessel, in a medium whose temperature did not exceed the sixth degree of Reaumur, was kept for eight days without sensibly losing its colour. But the vessel being uncorked, and left to the influence of the air in a higher temperature, its colour was considerably weakened, and in the space of twenty-four hours the urine was entirely discoloured, and acquired an ammoniacal odour. But, by continuing under the same influences, it presented the same phenomena already described, that is, it recovered its blue colour and a sour smell, slightly alcoholic, one may then deduce the same consequences. Another portion of the blue urine not altered, and to which there had been added some centiemes of sulphuric acid, left to the action of the air, under a temperature of from 13° to 16°, kept its colour for fifteen days, without disengaging any ammoniacal odour.

This experiment proves that the presence of the sulphuric acid opposed the so easy decomposition of the urine, and the consequent production of ammonia, and that even when any was formed, it must be entirely saturated by the sulphuric acid, with which it was brought into contact, and thus prevent the alteration of the blue colour.

The urine in question, a little time after being voided, did not produce any sensible change either on turmeric paper, nor on that of turnesol, that is, it manifested neither alcaline nor acid properties.

Sulphuric, nitric, muriatic, and acetic acids, poured on this urine, in a quantity sufficient to communicate to it well-marked characters of acidity, did not sensibly diminish its blue colour. Even chlorine added in a small proportion did not produce in it any effect.

On the contrary, with the alkalies, that is to say, with potass and ammonia, all the blue colour was destroyed, and the liquor assumed a yellow colour, similar to that of common urine, and of that which having been at first blue became yellow by the action of ammonia, the result of the spontaneous decomposition, as we have already shewn. On pouring the above-mentioned acids in sufficient quantity on the urine made yellow by potass or ammonia, the blue colour immediately re-appeared, an effect which also took place by the contact of the same acids with the urine made yellow by spontaneous decomposition, that is, by the action of the ammonia produced in the urine in this circumstance.

The blue urine, before being altered, when subjected to the action of the fire, and carried nearly to boiling, scarcely diffused the odour peculiar to ordinary urine, but there was distinctly perceived that of a solution of boiling sugar: this odour became more and more perceptible, in proportion as the liquor became concentrated, and particularly when it approached the consistence of a syrup. During this process the blue colour was not sensibly changed.

The residue arising from the evaporation of the urine, being carefully examined, presented the principles belonging to urine in the natural state: but a comparison being made of the proportions, the latter were found in infinitely smaller quantities in the urine now in question: the urea and uric acid existed in it in very small quantities: instead of it there was in it prussiate of iron, and a saccharine matter similar to that afforded by the urine in *diabetes mellitus.*

A small portion of the same residue being thrown on burning charcoal diffused an ammoniacal odour, but that of burned sugar was particularly distinguished in it. Another portion of the same residue being distilled in a glass retort, afforded slight traces of subcarbonate of ammonia, but more particularly the products yielded by vegetable substances, when treated over fire in close vessels.

The carbonized matter which remained in the retort was subjected to the action of muriatic acid: the solution being filtered and tried with prussiate of potass, and with infusion of galls, yielded with the first re-agent a deep blue colour, and was sensibly blackened by the second, which proves that the quantity of prussian blue contained in this particular urine was considerable. The different experiments just now described clearly prove in this secretion the simultaneous presence of prussian blue and of saccharine matter. However, to remove all doubt, I took another portion of the syrupy residue of this urine, and I diluted it in a sufficient quantity of distilled water; by rest there was precipitated a powder of a blue colour, which being separated from the liquor by decantation, and well washed, acquired a purer blue; when afterwards subjected to the action of the alkalies, acids, chlorine, and other re-agents, it gave precisely the same results as the prussiate of iron.

This being done, I poured into the supernatant liquor some subacetate of lead in a slight excess: by this means all the organic substance was precipitated, except the saccharine matter which remained in solution in the water; then there was made to pass through the filtered liquor a current of sulphuretted hydrogen in order to precipitate all the oxide of lead: the liquor again filtered was evaporated to the consistence of a thick syrup; there was then obtained a substance of a white yellow colour, of a well-marked sweet taste, which, when put on burning charcoal, diffused the odour of burned sugar: and treated with nitric acid presents all the phenomena and all the results which saccharine matter generally presents with the same agents. It appears to me that from all these results we may deduce the following corollaries:

1. That this urine contains prussian blue, and a saccharine matter, similar to that met with in the urine of *diabetes mellitus.*

2. That the blue colour of urine may also depend sometimes on the presence of another substance discovered by Braconnot, or designated by him under the name of *cyanurine:* but that it is without good reason that this learned chemist raises doubts with respect to the discovering of prussiate of iron in blue urine, a fact announced by Julia-Fontanelle, in the *Archives Génerales de Medicine for the year* 1823.

3. That probably the extraordinary presence of these substances, that is, the prussiate of iron and saccharine matter, may develope itself anew by reason of the *innormality* (*innormalité*) of the secreting function of the kidneys, which, according to the experiments of Wollaston, may be regarded in some measure as a chemico-galvanic process.

4. That free prussic acid having been discovered by Brugnatelli in the urine of a dropsical patient, prussiate of iron by Fourcroy in the blood of an hysterical woman, prussiate of iron in the urine by Moyon and by Julia-Fontanelle; that a similar blue matter having been observed by Reisel in the sputa of a woman affected with pneumonia accompanied with frequent vomiting: that the same phenomenon having presented itself to Dolxi, to Mogi, and even to

Julia-Fontanelle, in the perspiration of other individuals labouring under nervous affections,—it seems reasonable to think, that in the animal economy, under a morbid condition, the prussic acid may be engendered more frequently than has been hitherto imagined: but that if the circumstances are rare in which this effect becomes the cause of serious disturbances in the system, this must be imputed to the presence of some bases capable of neutralizing it, and of paralysing its deleterious effects.

5. That in comparing the symptoms and organic lesions which present themselves in cases of poisoning by prussic acid, and in the cholera morbus of India, it appears to me no abuse of analogy to state the opinion, with other physicians, that prussic acid performs some part in this terrible malady. This idea moreover is in accordance with all that has been hitherto written by Rossi on miasmus, and even on the Indian cholera.—*Journal de Pharmacie*, April, 1833. (Extract from the Memoirs of the Royal Academy of Turin.)—*Dublin Journal of Med. Science.*

### EXPERIMENTS ON HEMLOCK AND HENBANE.

Prof. Geiger, of Heidelberg, whilst recently engaged in making chemical experiments, succeeded in establishing some remarkable illustrations of the active principle of hemlock. Its base is an organic salt, which opens an entirely novel series of these highly interesting organic substances, for it is volatile, and similar to a volatile oil. The peculiar qualities of this substance, both intrinsically, and when brought into combination with acids, its rapidly changeable character, and the brilliant play of colours which it exhibits whilst undergoing change, render it one of the most interesting productions in organic chemistry. Its poison is of the deadliest description. The smallest quantity, applied inwardly, produces paralysis; and one or two grains are sufficient to kill the largest animal. Another of Professor Geiger's late discoveries is the active principle of henbane, (*atropin;*) its base is likewise an organic salt, but it is tenacious, admits of being reduced to a crystal, forms a crystalline salt with acids, like hemlock, and has a disagreeable smell, though it is not volatile, unless it be subjected to decomposition. Its poison is quite as deadly as that of the former, but exhibits dissimilar appearances, and is not so rapid in its effects. Animals, where even a minute dose is administered, become languid, cannot stand upon their legs, are attacked by convulsions, and die within six hours. The effect of this poison in dilating the pupil of the eye is extremely remarkable. The minutest portion of it, when applied to the eye of a cat, produces a dilatation of the pupil for the next four and twenty hours; and the hundreth part of a grain prolongs the appearance for the next seven or eight days, besides inducing other singular symptoms of poisoning.—*Rep. Pat. Invent. March*, 1833.

MARCHANTIA POLYMORPHA.

(760.) *Marchantia polymorpha* has lately been rendered peculiarly interesting in a physiological point of view, by Mirbel having shewn, in a memoir just published, that certain organs called *Stomata* exist in this plant, which were previously denied to be present in any of the mosses or their allies, indeed in any vegetables lower in the scale of creation than the ferns. He has, however, proved that they not only do exist, but that they exist in perfection; and, in tracing their evolution, he has thrown much very important light upon an obscure branch of vegetable structure. Mr. Griffith has likewise found Stomata in *Targiona hypophylla*. —*Prof. Burnett's Outlines of Botany*.

ARROW ROOT.

"Arrow root," says Berzelius, "being thought strengthening by some physicians, is sold very dear, for which reason it has been attempted to distinguish it with certainty from other kinds of starch. According to Guibourt it can be recognised, under the microscope, by the grains of arrow root being transparent, and smaller than those of potato starch, though their shape and size are variable also." Though I cannot help congratulating Berzelius on his newly-born tolerance of microscopic observations, still I must lament the complaisance which induces him to register, in catalogues invested with the authority of his name, such superficial observations as those which he takes from Gnibourt. According to the characters attributed by this writer to the fecula of arrow root, there are perhaps a hundred vegetables in France, whose fecula might be confounded with this Brazilian substance. What fecula is not transparent? And what fecula is more transparent than that of the solanum? Moreover, what fecula, with the exception of the fecula of chara seeds, has not smaller grains than the fecula of potatoes, and a size quite as variable? As to shapes, how many there are whose shapes are infinitely varied! But, by an unlucky chance, it happens, that so far from being transparent, the grains of arrow root are more shadowed than any that we have observed, and present characters which we have not met with in any other. These marks of distinction are as follows: The fecula of arrow root, when examined in large quantities, has a crystalline yet faint lustre; it is rougher to the touch than that of potatoes, and almost as much so as that of wheat starch; it contains small clots which resist pressure, and crackle under the fingers. When examined in water, and by the microscope, it presents groups of five or six, and even of ten or twelve grains, which the most rapid movement, and the most prolonged shaking, do not succeed in dissevering, but which continue to float over the liquid in company.

But the most distinctive of all the physical characters of this fecula is, that each grain is the half, or quarter, or third, &c. of a solid sphere; that others are small cylinders, with one extremity rounded *en calotte*, and the other flattened; lastly, that others exactly resemble a painter's *muller*: so that each of these grains has one or more angular surfaces, whose refraction produces those strong and varied shadows which we observe on the contour of the microscopic image; one might sometimes suppose one's self to be looking at crystals. Their structure is such, that it may be better known from a written description, than from the most exact drawing. Moreover, one often sees, through their transparent side, black lines crossing one another, sometimes like a T, and sometimes like a star, just as in the fecula of rye; and if we make the grains turn round, by moving the water, we can assure ourselves that these lines are by no means superficial, but, on the contrary, exist in the very heart of the grain, indicating the existence of cells, like those which I have observed in the lentil; the largest grains do not exceed $\frac{1}{25}$ of a millimetre in diameter. The adhesiveness of a great number of these grains to one another, and the angular surfaces which they have contracted by their agglutination, (always preserving, however, one of their curved surfaces,) would lead one to suppose that this fecula, which is composed of round and softish grains, has been treated, immediately after its extraction, by a violent stove-heat. What confirms me in this supposition is, that the long boiling, which is sufficient to spread out the integuments of potato fecula, so as to make them acquire from twenty to thirty times their original diameter, barely quadruples the volume of the grains of arrow root. This explains how it is that Pfaff found that ten grains of arrow root boiled in an ounce of water merely produce a mucilaginous liquid, while the same quantity of common fecula, in the same quantity of water, forms a gelatinous mass, a real starch.—*Nouveau Système de Chimie Organique, par F. V. Raspail*, § 3.

### THE ITCH.

The cause of this cutaneous eruption was long attributed to the presence and the bite of an insect, which, however, was sought for in vain in the pustules of itchy subjects at Paris. In 1812, Galès, in an *ex professo* thesis, declared that he had discovered this insect more than two hundred times in patients of the Parisian hospital. He caused a very handsome plate to be engraved of it, and even showed it alive to a committee of physicians and naturalists. Since that epoch no one has been so fortunate as Galès. This was not astonishing; for, as I demonstrated in 1829, the memoir of Galès was a mere hoax; and, instead of the itch-insect, Galès had submitted the insect of flour and cheese to the inspection of the committee. Nevertheless, in the treatise which I then published, to refute the memoir of Galès, (which had become quite classical,) I laid it down as a principle, that the hoax practised by Galès ought not to make us conclude that the insect, which the most conscientious observers had pointed out in itch, did not exist; and I added, that in some other climate, and perhaps in a different kind of itch, it might be possible to find it. The question was in this state when I had an opportunity of examining the itch of horses. I found the insect which Degéer had described, and which he had figured, though coarsely. As this question has acquired a certain importance in medicine, I have drawn this parasitic insect, with all the details I could observe, magnified one hundred times in diameter. The insect of the itch has a white, shining body, and its paws, as well as its snout, are purplish, when seen by reflected light; by refracted light its snout and paws are transparent and yellowish. But, what makes an enormous difference between the two insects is, that in the flour insect, (the *sarcopte* of Galès,) the eight paws are united round a breast-plate placed upon the thorax, while, in the insect of the horse-itch, the two front pair are pressed against the head, and, together with it, form a sort of fan, while the other two pair are separated from the first two by a very considerable space, and inserted, two by two, on each side of the abdomen. The paws, and the head especially, come forth from so many sheaths, which give the general form of the body the appearance of certain flattened and foliaceous shell-fish, or that of some fish-scales.—*Nouveau Système de Chimie Organique, par F. V. Raspail*, § 1321.

### LONGEVITY OF AUTHORS.

| | *Natural Philosophers.* | | *Poets.* | | *Moral Philosophers.* | |
|---|---|---|---|---|---|---|
| | Name. | Age. | Name. | Age. | Name. | Age. |
| 1 | Bacon, R. | 78 | Ariosto | 59 | Bacon | 65 |
| 2 | Buffon | 81 | Burns | 38 | Bayle | 59 |
| 3 | Copernicus | 70 | Byron | 37 | Berkely, G. | 79 |
| 4 | Cuvier | 64 | Camoens | 55 | Condorcet | 51 |
| 5 | Davy | 51 | Collins | 56 | Condillac | 65 |
| 6 | Euler | 76 | Cowley | 49 | Descartes | 54 |
| 7 | Franklin | 85 | Cowper | 69 | Diderot | 71 |
| 8 | Galileo | 78 | Dante | 56 | Ferguson, A. | 92 |
| 9 | Halley, Dr. | 86 | Dryden | 70 | Fichte, J. T. | 52 |
| 10 | Herschel | 84 | Goldsmith | 44 | Hartley, D. | 52 |
| 11 | Kepler | 60 | Gray | 57 | Helvetius | 57 |
| 12 | La Lande | 75 | Metastasio | 84 | Hobbes | 91 |
| 13 | La Place | 77 | Milton | 66 | Hume | 65 |
| 14 | Lowenhoeck | 91 | Petrarch | 68 | Kant | 80 |
| 15 | Leibnitz | 70 | Pope | 56 | Kaimes | 86 |
| 16 | Linnæus | 72 | Shenstone | 50 | Locke | 72 |
| 17 | Newton | 84 | Spenser | 46 | Malebranche | 77 |
| 18 | Tycho Brahe | 55 | Tasso | 52 | Reid, T. | 86 |
| 19 | Whiston | 95 | Thomson | 48 | Stewart, D. | 75 |
| 20 | Wollaston | 62 | Young | 84 | St. Lambert | 88 |
| | Total | 1494 | Total | 1144 | Total | 1417 |

| | *Authors on Law and Jurisprudence.* | | *Dramatists.* | | *Miscellaneous and Novel Writers.* | |
|---|---|---|---|---|---|---|
| | Name. | Age. | Name. | Age. | Name. | Age. |
| 1 | Bentham | 85 | Alfieri | 55 | Cervantes | 70 |
| 2 | Blackstone | 57 | Corneille | 78 | Le Sage | 80 |
| 3 | Butler, C. | 83 | Goethe | 82 | Scott | 62 |
| 4 | Coke | 85 | Massinger | 55 | Fielding | 47 |
| 5 | Erskine | 73 | Marlow | 32 | Smollett | 51 |
| 6 | Filangieri | 36 | Otway | 34 | Rabelais | 70 |
| 7 | Gifford | 48 | Racine | 60 | Defoe | 70 |
| 8 | Grotius | 63 | Schiller | 46 | Ratcliffe | 60 |
| 9 | Hale | 68 | Shakspeare | 52 | Richardson | 72 |
| 10 | Holt | 68 | Voltaire | 84 | Sterne | 56 |
| 11 | Littleton | 75 | Congreve | 59 | Johnson | 75 |
| 12 | Mansfield | 88 | Colman, G. | 61 | Addison | 48 |
| 13 | Montesquieu | 66 | Crebillon | 89 | Warton | 78 |
| 14 | Redesdale | 82 | Cumberland | 80 | Steele | 59 |
| 15 | Romilly | 61 | Farquhar | 30 | Tickell | 54 |
| 16 | Rolle | 68 | Goldoni | 85 | Montaigne | 60 |
| 17 | Tenterden | 78 | Jonson, B. | 63 | Bathurst, R. | 84 |
| 18 | Thurlow | 74 | Lope de Vega | 73 | Thornton | 44 |
| 19 | Vatel | 53 | Moliere | 53 | Hawkesworth | 59 |
| 20 | Wilmot | 83 | Murphy | 78 | Hazlitt | 58 |
| | Total | 1394 | Total | 1249 | Total | 1257 |

| | *Authors on Revealed Religion.* | | *Authors on Natural Religion.* | | *Medical Authors.* | |
|---|---|---|---|---|---|---|
| | Name. | Age. | Name. | Age. | Name. | Age. |
| 1 | Baxter | 76 | Annett | 55 | Brown | 54 |
| 2 | Bellarmine | 94 | Bolingbroke | 79 | Corvisart | 66 |
| 3 | Butler, John | 60 | Cardan | 75 | Cullen | 78 |
| 4 | Bossuet | 77 | Chubb | 65 | Darwin | 72 |
| 5 | Calvin | 56 | Drummond, Sir W. | 68 | Fordyce | 67 |
| 6 | Chillingworth | 43 | Dupuis | 67 | Fothergill | 69 |
| 7 | Doddridge | 54 | Freret, N. | 61 | Gall | 71 |
| 8 | Fox, G. | 67 | Gibbon | 58 | Gregory, John | 48 |
| 9 | Knox, John | 67 | Herbert, Lord | 68 | Harvey | 81 |
| 10 | Lowth | 77 | Jacobi | 56 | Heberden | 92 |
| 11 | Luther | 63 | Paine | 72 | Hoffman | 83 |
| 12 | Massillon | 79 | Pomponatius | 63 | Hunter, J. | 65 |
| 13 | Melancthon | 64 | Rousseau | 66 | Hunter, W. | 66 |
| 14 | Paley | 63 | Spinoza | 45 | Jenner | 75 |
| 15 | Porteus | 77 | St. Pierre | 77 | Mason Good | 64 |
| 16 | Priestley | 71 | Shaftesbury | 42 | Paracelsus | 43 |
| 17 | Sherlock | 67 | Tindal | 75 | Pinel | 84 |
| 18 | Wesley | 88 | Toland | 53 | Sydenham | 66 |
| 19 | Whitfield | 56 | Vanini | 34 | Tissot | 70 |
| 20 | Wycliffe | 61 | Volney | 66 | Willis, T. | 54 |
| | Total | 1350 | Total | 1245 | Total | 1368 |

| | *Artists.* | | *Philologists.* | | *Musical Composers.* | |
|---|---|---|---|---|---|---|
| | Name. | Age. | Name. | A g | Name. | Age. |
| 1 | Bandinelli | 72 | Bentley | 81 | Arne | 68 |
| 2 | Bernini | 82 | Burton | 64 | Bach | 66 |
| 3 | Canova | 65 | Casaubon | 55 | Beethoven | 57 |
| 4 | Donatello | 83 | Cheke | 44 | Burney | 88 |
| 5 | Flaxman | 71 | Hartzheim | 70 | Bull | 41 |
| 6 | Ghiberti | 64 | Harman, J. | 77 | Cimarosa | 41 |
| 7 | Giotto | 60 | Heyne | 84 | Corelli | 60 |
| 8 | Michael Angelo | 96 | Lipsius | 60 | Gluck | 75 |
| 9 | San Sovino | 91 | Parr | 80 | Gretry | 72 |
| 10 | Verocchio | 56 | Pauw | 61 | Handel | 75 |
| 11 | Caracci, A. | 49 | Pighius | 84 | Haydn | 77 |
| 12 | Claude | 82 | Porson | 50 | Kalkbrenner | 51 |
| 13 | David | 76 | Raphelengius | 59 | Keiser | 62 |
| 14 | Guido | 67 | Salmatius | 66 | Martini | 78 |
| 15 | Raphael | 37 | Scaliger, J. J. | 69 | Mozart | 36 |
| 16 | Reynolds | 69 | Sigonius | 60 | Paisello | 75 |
| 17 | Salvator Rosa | 58 | Stephens, H. | 71 | Piccini | 71 |
| 18 | Titian | 96 | Sylburgius | 51 | Porpore | 78 |
| 19 | Veronese, Paul | 56 | Vossius | 73 | Scarlatti | 78 |
| 20 | West | 82 | Wolfius | 64 | Weber | 40 |
| | Total | 1412 | Total | 1323 | Total | 1289 |

*Madden's Infirmities of Genius.*

## Figures and Tables

**Figure f1:**
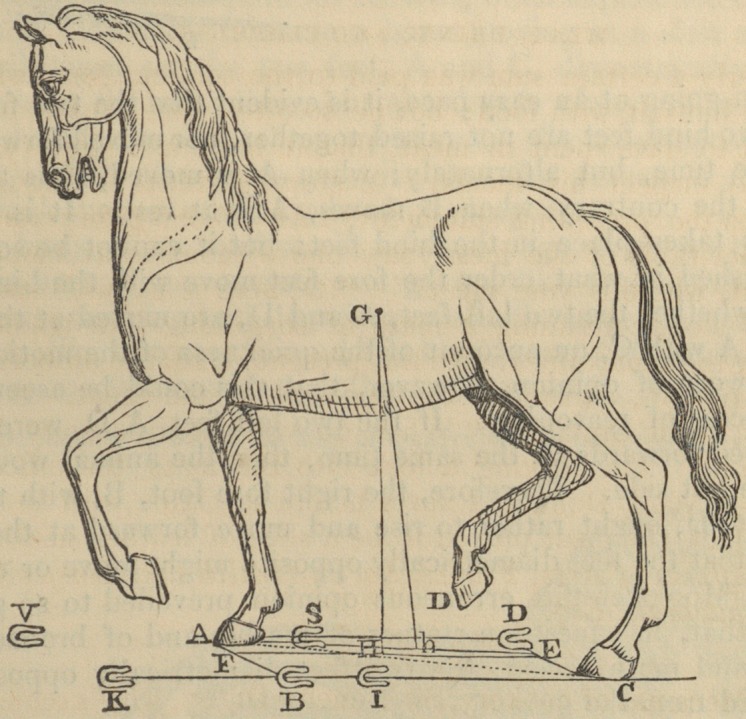


**Figure f2:**
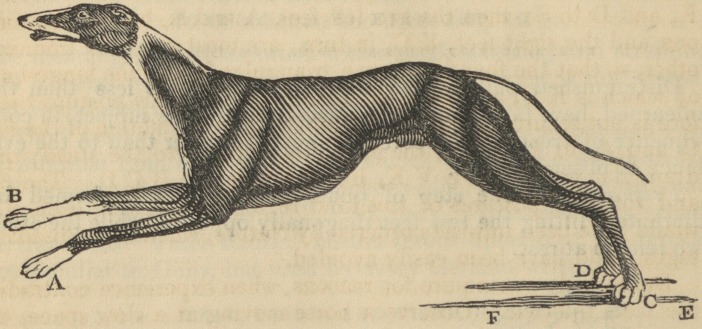


**Figure f3:**
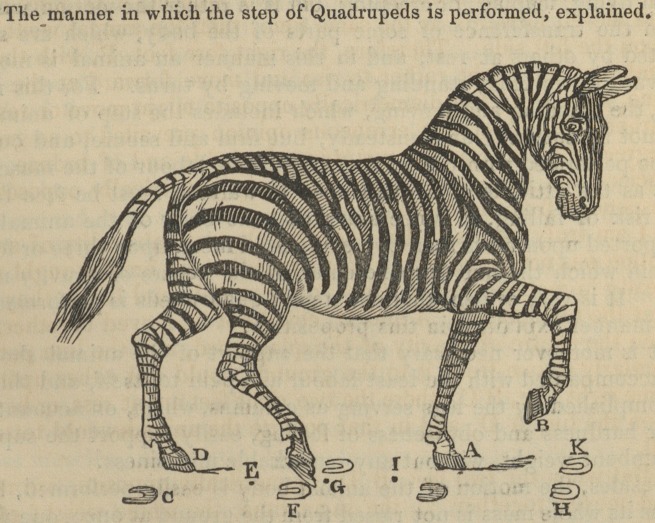


**Figure f4:**
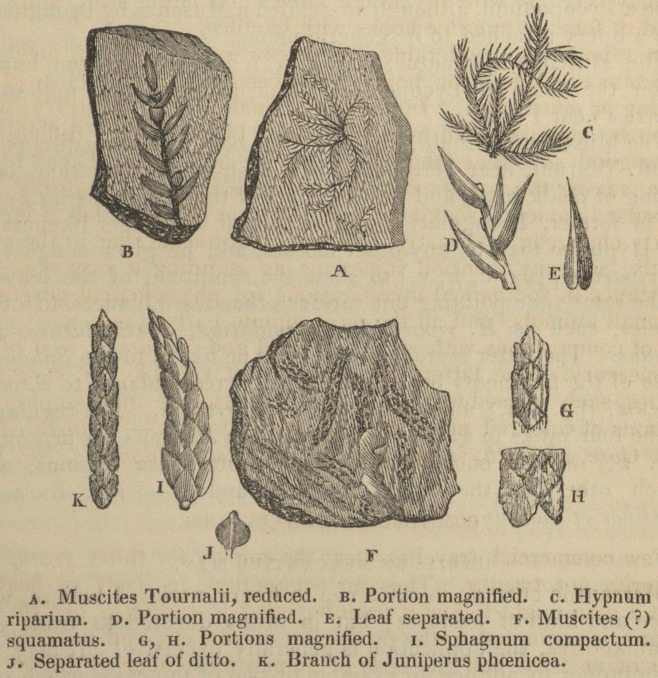


**Figure f5:**